# Identification and analysis of lncRNA, miRNA and mRNA related to subcutaneous and intramuscular fat in Laiwu pigs

**DOI:** 10.3389/fendo.2022.1081460

**Published:** 2023-01-13

**Authors:** Hui Feng, Tianyi Liu, Salsabeel Yousuf, Xiuxiu Zhang, Wanlong Huang, Ai Li, Lingli Xie, Xiangyang Miao

**Affiliations:** Institute of Animal Sciences, Chinese Academy of Agricultural Sciences, Beijing, China

**Keywords:** lncRNA, co-expression, ceRNA network, intramuscular fat, adipogenesis

## Abstract

**Background:**

Long non-coding RNAs (lncRNAs) regulate adipocyte differentiation and metabolism, However, their function on subcutaneous and intramuscular adipose tissues in pigs is unclear. Intramuscular fat (IMF) is an important indicator for evaluating meat quality. Breeds with high IMF content are often accompanied by high subcutaneous fat (SCF), which severely affects the meat rate of pigs. It is of great significance for porcine breeding to study the mechanism of lncRNA related to adipogenesis and lipid metabolism.

**Methods:**

We identified differentially expressed lncRNAs, miRNAs and mRNAs in subcutaneous and intramuscular adipose tissues in three female Laiwu pigs by deep RNA-sequencing(|log2foldchange|≥1, *P*_value *≤* 0.05). The gene expression profiles of IMF and SCF in Laiwu pigs were comparatively analyzed by Bioinformatics methods to identify key lncRNAs, miRNAs, and mRNAs associated with lipid metabolism and adipogenesis.

**Results:**

A total of 1209 lncRNAs (DElncRNAs), 286 miRNAs (DEmiRNAs), and 1597 mRNAs (DEgenes) were differentially expressed between two types of adipose. Among them, 17 DElncRNAs and 103 target genes play a role in the co-expression network, as well as 59 DElncRNAs, 44 DEmiRNAs, and 88 DEgenes involved in ceRNA network. In GO(Gene Ontology) and KEGG (Kyoto Encyclopedia of Genes and Genomes) pathway analysis of DElncRNAs their target genes involved in many adipogenesis and lipid metabolism biological processes and signaling pathways, such as PPAR signaling pathway, Wnt signaling pathway, MAPK signaling pathway.

**Conclusions:**

By constructing co-expression and ceRNAs network we found that Wnt signaling pathway play a critical regulatory role in intramuscular adipogenesis and lipid accumulation in Laiwu pigs. TCONS_00006525, TCONS_00046551 and TCONS_00000528 may target WNT5A, WNT10B and FDZ3 in co-expression network, TCONS_00026517 and other lncRNAs regulate the expression of PPARG, RXRG and SCD in ceRNA network, and were involved in Wnt signaling pathway. This study provides a theoretical basis for further understanding the post-transcriptional regulation mechanism of meat quality formation, predicting and treating diseases caused by ectopic fat.

## 1 Introduction

Intramuscular fat content is closely related to meat quality. Subcutaneous fat is the largest fat depot, and generally has a significant negative correlation with leanness of livestock. The goal of livestock breeding production is high leanness and fine meat quality, This should increase intramuscular fat and reduce subcutaneous fat in livestock. However, the deposition of intramuscular fat and subcutaneous fat is synergistic. Fat is first deposited under the skin to a certain extent and then starts to be deposited in the intramuscular and visceral areas. Fat distribution is closely related to disease, and ectopic fat deposition is often more disease-causing than subcutaneous fat (1). Increased intramuscular fat leads to decreased skeletal muscle regeneration, muscle atrophy, and even skeletal muscle-related diseases, promoting proliferation and differentiation of myofiber-adipose-derived progenitor cells (FAPs) to adipocytes after muscle injury (2). Decreased muscle strength of the summer solstice in type 2 diabetic chemistry is also associated with excessive ectopic fat deposition, especially intramuscular fat deposition (3, 4). The breeding goal for livestock product production is to balance the relationship between meat quality and disease, and to control the content of intramuscular fat deposits.

lncRNAs are non-coding RNAs longer than 200 bp. lncRNA research has led to the discovery of a variety of lncRNAs capable of participating in lipogenic differentiation and lipid metabolism. The steroid receptor RNA activator 1 (SRA1) lncRNA binds to and coactivates PPARγ, which was the first lncRNA found to be involved in adipocyte development (5). Mice knocked out of the SRA1 locus were able to alleviate obesity and glucose intolerance induced by a high-fat diet and increase systemic insulin sensitivity (6). Many experiments verified that lncRNAs are differentially expressed during adipogenesis. FIRRE transcription sites play regulatory roles in adipogenesis (6). Bmncr affects the local chromatin spatial structure and transcription of fibromodulin (FMOD), promotes phospholipid-lysophospholipid transacylase (Tafazzin, TAZ) and c-abl cancer gene 1, non-receptor tyrosine kinase (c-abl oncogene 1, non-receptor tyrosine kinase, ABL1) interactions, and inhibits differentiation of bone marrow mesenchymal stem cells to adipocytes (7). Linc-ADAL interacts with heterogeneous nuclear ribonucleoprotein U (hnRNPU) and insulin-like growth factor 2 mRNA binding protein 2 (IGF2BP2) to regulate adipocyte differentiation and adipogenesis (8). AC092159.2 is able to activate TMEM18 and promote adipocyte differentiation (9). LncRAP2 is a conserved cytoplasmic lncRNA that binds to mRNA translation regulatory proteins to form the lncRAP2-Igf2bp2 complex, which selectively binds adipogenic regulators and energy expenditure factors to promote adipogenesis and energy expenditure (10). lncRNA-PCAT1 inhibits the differentiation of human adipose stem cells (hADSCs) toward adiposity by targeting the miR-145-5p-TLR4-Toll-like receptor (TLR) signaling pathway (11). LncRNA-NEF inhibits the differentiation of adipose-derived stem cells (ADSCs) to adipocytes by regulating the miR-155-PTEN axis (12). LncSAMM50 is highly expressed in the antisense chain of the upstream region of SAMM50 in adipose tissue, and promotes proliferation and differentiation of adipocytes by upregulation of PPARG and C/EBPα expression without affecting SAMM50 expression (13). Lnc-ORA is a newly identified obesity-associated lncRNA. lnc-ORA is significantly increased in obese mice. When lnc-ORA was knocked down, the mRNA and protein expression levels of cell cycle marker genes were reduced. This affects the PI3K/AKT/mTOR signaling pathway and inhibits adipocyte proliferation and differentiation (14). LIPE-AS1 is also a potential regulator of adipogenesis promotion. When LIPE-AS1 expression was inhibited, the expression of major adipogenic factors, including PPARG and CEBPA, was downregulated. This results in a significant decrease in OP9 precursor adipocyte differentiation and even to apoptosis (15). Gm15290 is one of the most upregulated lncRNAs in obese mouse adiposity. Gm15290 acts as a molecular sponge for miR-27b, involved in the regulation of genes such as PPARγ, C/EBPα and aP2, increasing lipid deposition (16). TUG1 reduced inflammation through miR-204/SIRT1 axis enhancing insulin sensitivity in white adipocytes When TUG1 was overexpressed, miR-204 expression was decreased and SIRT1, p-AKT, glucose transporter 4 (GLUT4) and peroxisome proliferator-activated receptor γ (PPARγ) expression was increased, accompanied by relief of weight gain, insulin resistance and inflammation triggered by high fat diet (17). VLDLR antisense RNA1 (VLDLR-AS1) also has the potential to regulate lipid metabolism. VLDLR-AS1 is a potential molecular sponge for miR-600, which regulates UCH-L1 involvement in lipid metabolism through the Wnt/β-catenin signaling pathway (18). LncRNA Blnc1 is one of the conserved lncRNAs in the process of adipocyte differentiation It can promote adipocyte metabolism by binding to protein Zbtb7b (19). With the research of lncRNAs combined with bioinformatics technology, more and more lncRNAs have been identified with potential functions to regulate adipocyte metabolism, such as lncRNA uc.333 (20), lncIMF2 (21), MALAT1 (22), lncPRDM16 (23), etc. However, there are some limitations for those studies. It is necessary to conduct more experiments to further validate the mechanism of lncRNA action in adipocytes. In previous study, we conducted the genome-wide analysis of lncRNAs in intramuscular fat of adipose and lean pigs, and identified lncRNAs with the potential to regulate intramuscular fat deposition and lipid metabolism by constructing co-expression networks (24). CeRNA regulates network mechanism, lncRNA can act as a molecular sponge for miR-128-3p, miR-27b-3p to increase the miRNA target gene PPARG and promote the differentiation of intra-muscular adipocyte (25). However, there are few studies on the differences between subcutaneous and intramuscular fat deposition. In addition, the key genes that promote intramuscular fat generally also promote subcutaneous fat deposition.

In this experiment, we selected intramuscular fat and subcutaneous fat from Laiwu pigs for differential lncRNA identification and bioinformatics analysis to study the relevant lncRNAs that promote intramuscular fat deposition without increasing or decreasing subcutaneous fat deposition. Furthermore, we explored the molecular mechanisms of differentially expressed lncRNAs, miRNAs and genes through Gene Ontology (GO), Kyoto Encyclopedia of Genes and Genomes (KEGG) pathway, co-expression network, ceRNA regulatory network and protein interactions network analysis to explore the molecular mechanisms of differentially expressed lncRNAs, miRNAs and genes. The results can provide effective lncRNAs for optimizing lipid deposition in pigs and molecular markers for the study of reducing ectopic lipid deposition and treating lipid metabolism-related diseases.

## 2 Materials and methods

### 2.1 The preparation of animal and adipose tissue samples

In this study, 3 healthy female Laiwu pigs (180 days old) bred under the same conditions by Daqian Husbandry Co., Ltd., Laiwu City, Shandong Province, China were used as experimental animals. Animals were slaughtered by exsanguinations, the subcutaneous adipose tissues (L_PX) were from back fat the 3-4th rib, and the intramuscular adipose tissues (L_JN)were from longissimus dorsi muscle at the same part. Tissues were collected into tubes and immediately frozen in liquid nitrogen, and then transferred to refrigerator at -80℃ for later use.

### 2.2 RNA extraction and RNA-seq

Total RNA was extracted from the fat using the TRIZOL RNA extraction kits. RNA concentration and purity were checked using a Nano Drop 2000 spectrophotometer (Thermo Fisher Scientific, Wilmington, DE). The OD260 nm/OD280 nm between 1.9-2.1indicated that the RNA concentration and purity were qualified and can be used for subsequent analysis. The quality of RNA was evaluated by Bioanalyzer 2100. The results of RIN ≥ 7 and 28S/18S ≥ 0.7, indicated that the total RNA was high-quality RNA. Finally, potential genomic DNA contamination was eliminated by RNase-free DNase. The qualified total RNA was taken to construct the long-chain RNA library and small RNA library respectively. We used Illumina HiSeqTM 2500 sequencing platform for sequencing. The raw reads was obtained through paired-end sequencing.

### 2.3 Reference genome mapping and transcriptome assembly

The reference genome and gene annotation information of pigs were obtained in ENSEMBL database (https://asia.ensembl.org/index.html). The clean reads were compared with the reference genome using Hisat2 software. The proportions of various alignment types are counted, and the alignment results are evaluated by the RSeqQC suite (26). StringTie software was used to splice from the reads aligned with the reference genome. Each separately assembled transcript was fused and spliced into a complete transcript, and the abundance of the transcript was estimated (27). We compared the spliced sequences with the transcripts of the reference gene one by one by using CuffCompare software, filtered the known non lncRNA sequences and other transcripts that are completely matched or similar to ncRNA and mRNA, and used class_ Code classification criteria determine the location and type of transcripts on chromosomes (28). CPC (29), CNCI (30), Pfam (31) and PLEK (32) were used to filter transcripts with coding potential in sequences longer than 200 bp.

### 2.4 Differential gene expression analysis

Bowtie2 (33) software was used to statistically analyze the sequence expression abundance, and the number of fragments per KB per million reads (FPKM) algorithm from a gene per million fragments was used to correct the gene expression level, so as to eliminate the influence of sequencing depth, gene length and sample difference on gene expression. The DEseq2 package in R language was used for differential expression analysis. | log2foldchange | ≥ 1 and P_ value ≤ 0.05 were used to screen differentially expressed genes (34).

### 2.5 Co-expression network analysis

As a non-coding RNA, lncRNA is mainly involved in post transcriptional regulation. At present, there are few existing lncRNA databases. The lncRNAs identified in this experiment were novel lncRNAs without symbol numbers. By analyzing lncRNA and mRNA co expression network, we predicted the cis and trans effects of lncRNA. The pierce correlation coefficient of lncRNA and mRNA expression were calculated. If P_value ≤ 0.05 and |correlation| ≥ 0.9, lncRNA and mRNA were correlated, and used to construct co-expression network. We searched all coding genes within 300K upstream and downstream of differentially expressed lncRNA, and took the genes co expressed with lncRNA as the target genes of cis- action of lncRNA. The minimum free energy of complementary pairing between lncRNA and mRNA was calculated by RNAplex software (35). If the minimum free energy < - 20, the predicted result was the best base pairing relationship.

### 2.6 CeRNA network analysis

We used miRanda to predict the binding relationship of lncRNA, mRNA and miRNA according to score≥150 and energy≤-30kcal/mol, and retained the differentially expressed miRNA-mRNA and miRNA-lncRNA pairs in IMF and SCF. We selected DElncRNAs and DEmRNAs that bind to the same miRNA to construct ceRNA network.

### 2.7 Functional enrichment and protein-protein interaction (PPI) network analysis

We used clusterProfiler package of R Studio to annotate target genes function, including GO enrichment and KEGG pathway (36). When P_value ≤ 0.05, GO terms and KEGG pathways were significantly enriched. According to STRING database (https://cn.string-db.org/), we performed PPI network analysis on the target genes to further study the interaction among target genes in IMF and SCF of Laiwu pigs. The PPI network data files were visualized using Cytoscape to identify key target genes (37).

### 2.8 Quantitative real-time PCR validation

To further verify the reliability of sequencing results, we randomly selected 12 differentially expressed genes(including 6 lncRNAs, 3 miRNAs and 3 mRNAs) for qRT-PCR verification. Total RNA was extracted from the IMF and SCF tissue samples with Total RNA Extraction Kit (DNase I) kit (Genepool, Cat# GPQ1801). 3 μL RNA was taken, and was electrophoretic with 1% agarose gel to detect the integrity of RNA. lncRNA cDNA Synthesis Kit (GenePool, Cat# GPQ1806) and miRNA cDNA Synthesis Kit (GenePool, Cat# GPQ1804) were used for reverse transcription. BIOER LineGene 9600Plus fluorescence quantitative instrument was used to perform the PCR analysis. All reactions were repeated in triplicate, and the relative quantitative expression levels were calculated by 2^-△△Ct^ method. GAPDH was reference gene of lncRNA and mRNA, and U6 was reference gene of miRNA. Different expression levels were determined using a T-test. **Table 1** shows the primer information.

**Table 1:** Genes and the corresponding primer sequences.

Gene	Forward primer	Reverse primer	size/bp
**UBA7**	ATGACCTGAAGTGGACCTGTTG	CGACCATCCTGCCGAGTAGA	158
**PPARD**	CGAGTTCGCCAAGAGCATCC	GCACGCCGTACTTGAGAAGG	77
**PODXL**	ACTCCTCCTGTTCCTGTGACT	TGGCGTTATTGTAATCAGCATCTC	147
**GAPDH**	TGAAGGTCGGAGTGAACGGATT	CCATGTAGTGGAGGTCAATGAAGG	120
**miR-296-5p**	GCCCCCCCCAATCCTGT		
**novel407_mature**	GTCCTCCAGGAGCCCACA		
**miR-125b**	TCCCTGAGACCCTAACTTGTG		
**U6-F**	CTCGCTTCGGCAGCACA	AACGCTTCACGAATTTGCGT	
**TCONS_00003355**	GGCAGAGCAGAAGAGTAATGGA	TGTATATGAAGAGGCAGAGGAAGG	110
**TCONS_00026517**	CCATCTCCTCGTATCCAAGTCTC	CATCCTGTGCGTCGTCTCAA	122
**TCONS_00056923**	CCGTGTGCCAGTTCCAGTT	AGACAGACCAAGATGAGCCTAAG	225
**TCONS_00000528**	ACACGCAGGCAACTAACTCA	GAATGAATGAAGACGACATCACTTG	145
**TCONS_00006525**	TTGCTACCATACATACACCATCTTG	TTGCTTCTTCCTTGGCTCAGT	196
**TCONS_00046551**	TCTGTTCATTAAGGTAGGCGTTAAG	TTAGCAGGAGGTTAGAGGATTGTAT	122

(Amplification cycle temperature: 95°C 30 sec, (95°C 5 sec, 60°C 30 sec) × 45).

## 3 Results

### 3.1 Reads mapping to the Laiwu pig transcriptome and quality control

**Figure 1:**
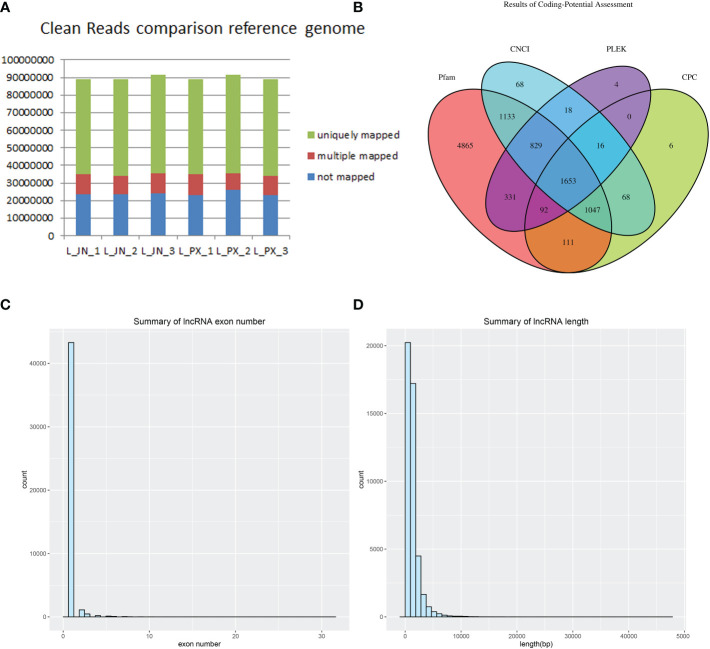
Long RNA comparison reference genome results **(A)** lncRNA database comparison results **(B)** The count of lncRNA exons **(C)** Transcript length distribution **(D)**.

Three Laiwu pigs with similar weight were selected for deep sequencing (clean reads≥10GB/sample) of intramuscular and subcutaneous adipose tissue. Clean reads were obtained from the original data after quality control. We compared clean reads with the reference genome of pigs, The results showed that 73% of the fragments in each sample were compared to the reference gene, and 12% of the reads had multiple alignment positions on the reference sequence (**Figure 1A**). The result of lncRNA was obtained by intersecting these transcripts through the predicted protein-coding ability of PLEK, CNCI, CPC and Pfam (**Figure 1B**). The lncRNAs were identified, the number of exons of the transcripts was predicted. The transcripts with the number of exons ≥2 (**Figure 1C**) and the length greater than 200bp were screened (**Figure 1D**).

### 3.2 Identification of differentially expressed genes

**Figure 2:**
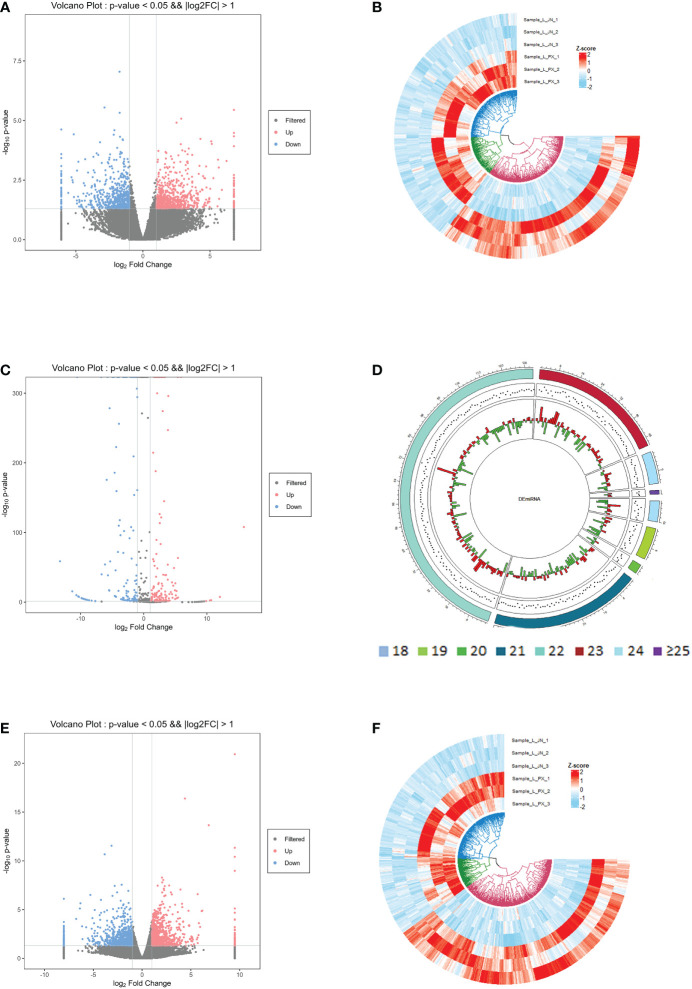
Differentially expressed gene information. Differentially expressed lncRNA volcano map **(A)** and heat map **(B)**; differentially expressed miRNA volcano map **(C)** and length distribution map **(D)**; differentially expressed mRNA volcano map **(E)** and heat map **(F)**.

In the differential expression analysis of lncRNA, miRNA and mRNA in subcutaneous and intramuscular adipose tissues of Laiwu pigs, we found that 1258 lncRNAs, 286 miRNAs and 1665 mRNAs differentially expressed in the two adipose tissues with p_value ≤ 0.05 and |log2foldchange|≥1 as screening conditions (**Figure 2**). Among them, there were 666 lncRNAs, 146 miRNAs and 777 mRNAs down-regulated expression and 592 lncRNAs, 140 miRNAs and 888 mRNAs up-regulated expression in the intramuscular adipose tissue. The differentially expressed lncRNAs included 948 lincRNAs, 285 inronic lncRNAs, 15 sense lncRNAs and 10 anti-sense lncRNAs. The differentially expressed miRNAs were mainly distributed in the length of 21-23 bp.

### 3.3 Co-expression network

**Figure 3:**
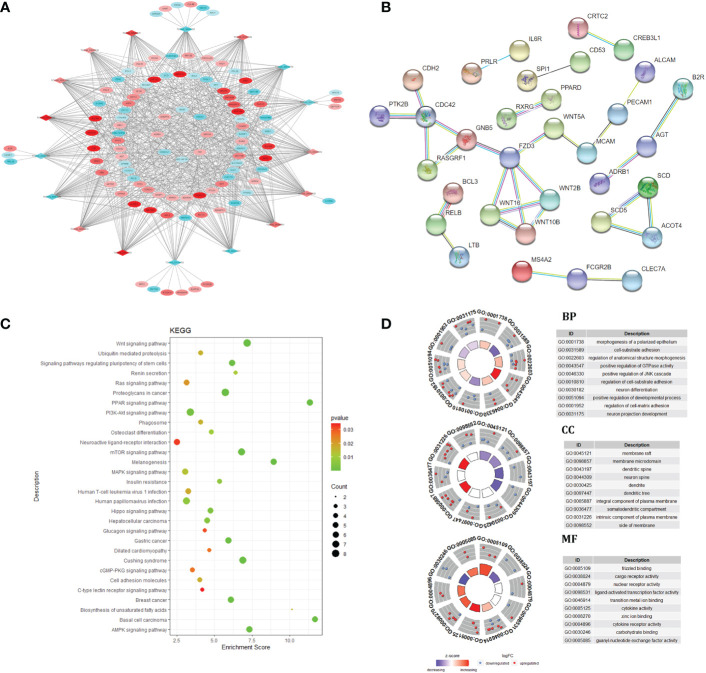
Co-expression network and target gene biology analysis. lncRNA and mRNA co-expression regulatory network **(A)**; protein-protein interaction network **(B)**; KEGG pathway enrichment analysis **(C)**; GO enrichment results **(D)**.

The lncRNA mRNA co expression network was constructed by calculating the Pearson correlation coefficient (|PCC| ≥ 0.9) of differentially expressed lncRNA and mRNA, t In intramuscular fat and subcutaneous fat, 17 DElncRNAs and 103 target genes played an important roles in the co expression network. GO and KEGG function enrichment analysis was carried out for the regulatory network. The results showed that the regulatory network caused differences in subcutaneous fat and intramuscular fat deposition by regulating PPAR signaling pathway, Wnt signaling pathway, Hedgehog signaling pathway, MAPK signaling pathway, Biosynthesis of unsaturated fat acids and other signal pathways. The target gene was used in PPI, and the PPI network center was taken. In **Figure 3**, this gene cluster was significantly enriched in Wnt signaling pathway. This may be a key regulatory signal pathway for the difference between intramuscular fat tissue and subcutaneous fat tissue.

### 3.4 The prediction of lncRNA binding protein

lncRNAs was often used as functional elements to regulate protein activity by binding to specific proteins. lncRNA and protein binding properties are calculated by RPISeq database. We took the best lncRNA and protein binding results for display. Usually, the average values of RF Classifier and SVM Classifier were greater than 0.5, lncRNA and protein have binding potential. TCONS_00006525 has the potential to bind more proteins and can be used as a key lncRNA for follow-up studies. The results are shown in **Table 2**.

**Table 2:** lncRNAs and proteins binding information.

Protein ID	RNA ID	RF Classifier	SVM Classifier
FOSL1	TCONS_00081807	0.9	0.988
FOSL1	TCONS_00046551	0.9	0.988
FZD3	TCONS_00006525	0.9	0.988
PPARD	TCONS_00006525	0.9	0.988
PRICKLE3	TCONS_00006525	0.95	0.992
WNT2B	TCONS_00006525	0.9	0.935
WNT5A	TCONS_00006525	0.9	0.939
WNT10B	TCONS_00006525	0.95	0.988
WNT16	TCONS_00006525	0.85	0.984

### 3.5 CeRNA networks between lncRNAs and target genes

**Figure 4:**
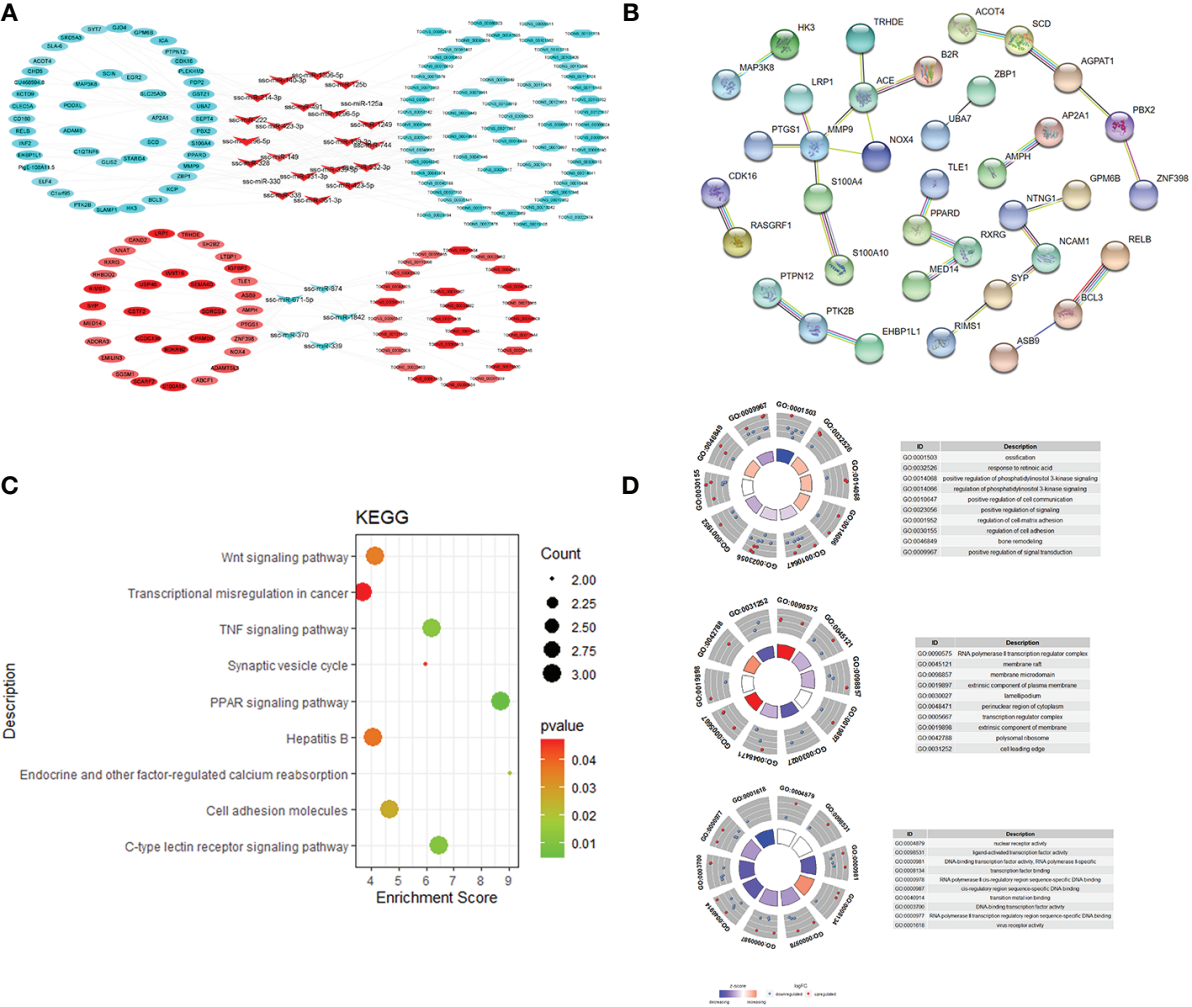
ceRNA regulatory network and target gene biology analysis of lncRNA, miRNA and mRNA regulatory network **(A)**; protein-protein interaction network **(B)**; KEGG pathway enrichment analysis results **(C)**; GO enrichment results **(D)**.

lncRNAs can act as molecular sponges for miRNAs, competitively binding miRNAs and inhibiting miRNA functions. The targeting relationships among lncRNA, miRNA and mRNA were predicted by applying miRanda to construct ceRNA regulatory network. ceRNA network contains 88 genes (40 up-regulated and 48 down-regulated), 44 miRNAs (29 up-regulated and 15 down-regulated), 59 lncRNAs (27 up-regulated and 63 down-regulated), of which the up-regulated expression of TCONS_00017887, TCONS_00056923, and TCONS_00022146, which are down-regulated lncRNAs, are in the critical nodes (**Figures 4A, B**). These critical node lncRNAs competitively bind ssc-miR-671-5p, ssc-miR-361-3p, ssc-miR-331-3p, potentially regulating RXRG, SCD, PPARD and etc. GO and KEGG functional enrichment analysis of the genes in ceRNA regulatory network showed that these genes were significantly enriched in PPAR signaling pathway, C-type lectin receptor signaling pathway, Wnt signaling pathway, and TNF signaling pathway (**Figures 4C, D**).

### 3.6 Wnt signaling pathways

**Figure 5:**
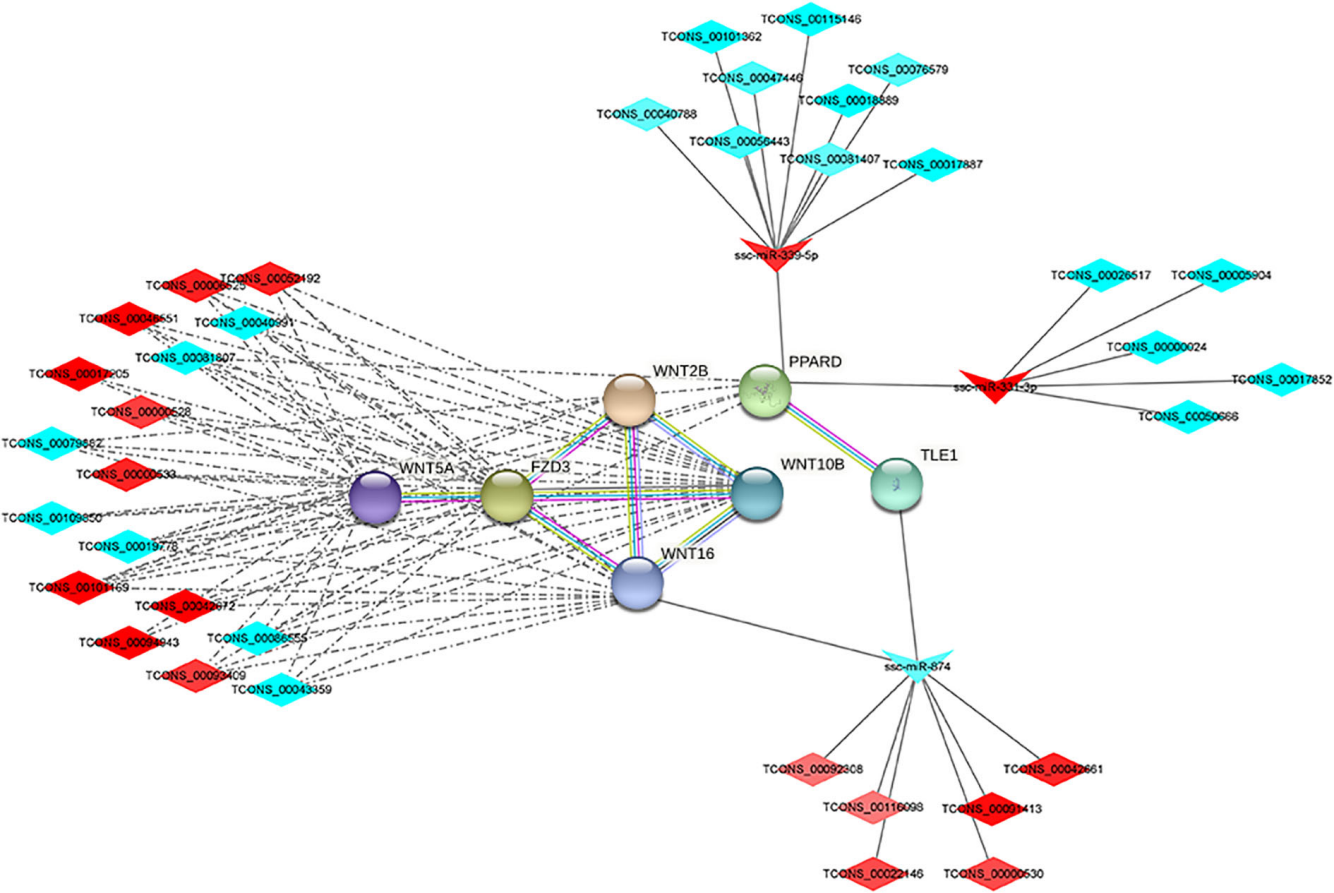
Regulatory network of Wnt signaling pathway.

Wnt signaling pathway-related differential mRNAs, lncRNAs and miRNAs were selected from the protein-protein interaction network through GO and KEGG pathway enrichment analysis. 9 differential genes were significantly enriched in the Wnt signaling pathway, including WNT5A, FZD3, WNT10B, WNT16, FOSL1, PPARD, PRICKLE3, WNT2B and TLE1. Among them, WNT5A, FZD3, WNT10B, WNT16, TLE1, PPARD and WNT2B were involved in protein-protein interactions. 10 up-regulated DElncRNAs and 7 down-regulated DElncRNAs were involved in protein-protein interactions through co-expression networks. 14 down-regulated DElncRNAs and 6 up-regulated DElncRNAs were involved in regulation through the ceRNA network (**Figure 5**). Among them, WNT5A, WNT2B, WNT10B, WNT16, PRICKLE3 and FZD3 were expressed at higher levels in IMF, PPARD was expressed at higher levels in SCF, and FOSL1. Although differentially expressed in the two adipose tissues, the expression levels were lower than other genes.

### 3.7 qRT-PCR validation

**Figure 6:**
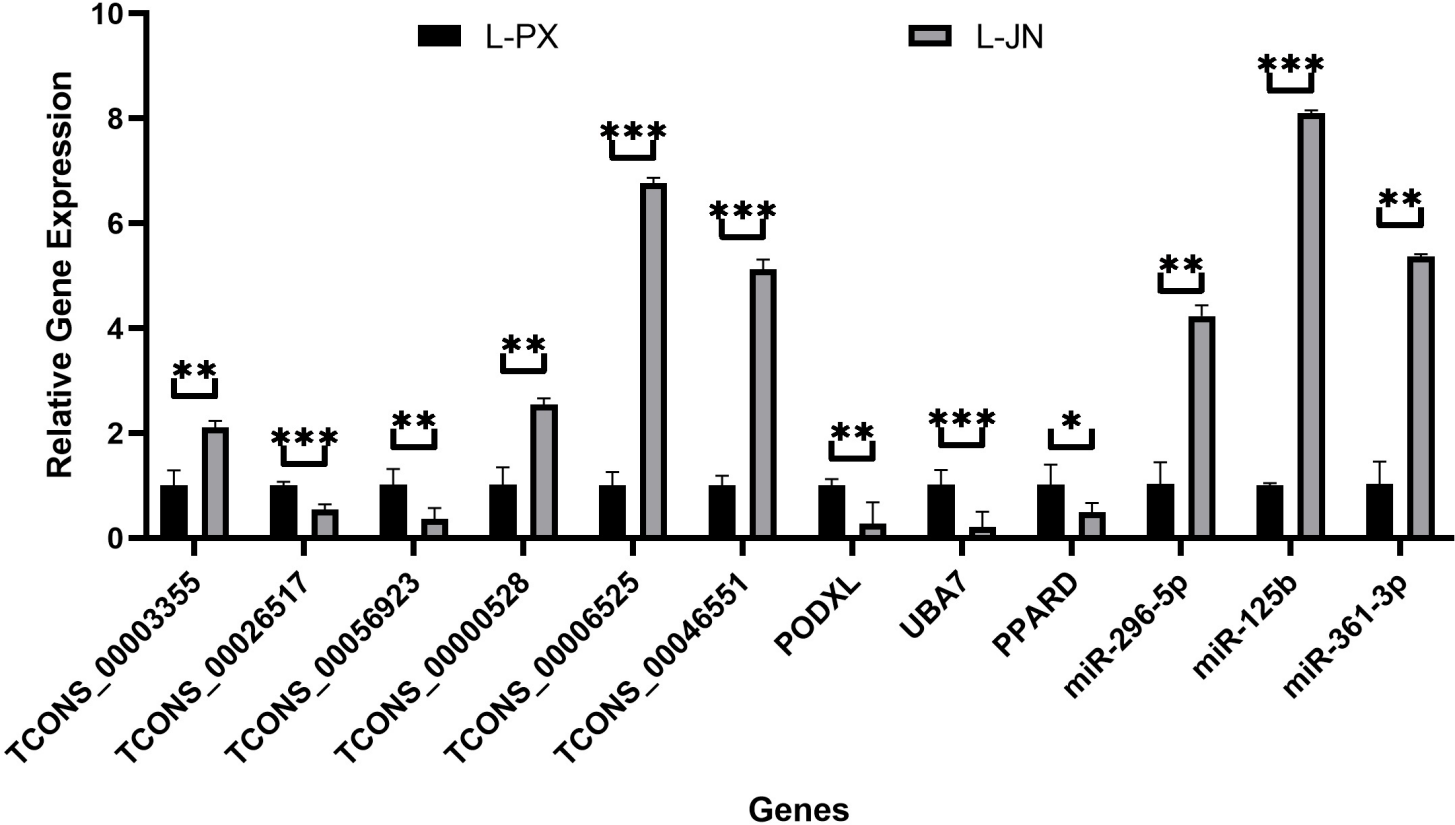
qRT-PCR validation results. The differential expression of genes between intramuscular and subcutaneous adipose tissue in Laiwu pig was verified qRT-PCR. *p < 0.05; **p < 0.01; ***p < 0.001.

12 differentially expressed RNA, including 6 lncRNAs, 3 miRNAs and 3 mRNAs, were randomly selected for verification. In **Figure 6**, the Q-PCR results show that TCONS_00003355, TCONS_00000528, TCONS_00006525, TCONS_00046551, miR-296-5p, miR-125b and miR-361-3p were significantly high expressed, and TCONS_00026517, TCONS_00056923, PODXL, UBA7 and PPARD were low expressed in intramuscular fat than that of subcutaneous fat. These data are consistent with the sequencing results, and the sequencing results were reliable.

## 4 Discussions

In this study, we screened lncRNAs differentially expressed in the two tissues by deep sequencing the transcripts of intramuscular fat and subcutaneous adipose tissue, and identified a total of 1258 DElncRNAs DElncRNA-mRNA co-expression network and ceRNA network analysis and its target gene function analysis showed that lncRNAs mainly regulate lipogenesis and lipid metabolism in IMF and SCF by regulating signaling pathways, biofilm composition, transcription factors. lncRNAs mainly regulate the biological processes such as signaling pathways, biofilm composition, transcription factor activity and Wnt, PPAR, TNF signaling pathways to regulate adipogenesis and lipid metabolism in IMF and SCF.

In the lncRNA-mRNA co-expression network, GO annotation analysis showed that the target genes were significantly enriched in biological functions, such as signaling pathway regulation, cell biofilm composition and enzyme activity regulation. In KEGG through-loop analysis the target genes were mainly involved in signaling pathways such as Wnt, PPAR and melanogenesis. In this study, 8 differential genes were significantly enriched in Wnt signaling pathway, including WNT5A, FZD3, WNT10B, WNT16, FOSL1, PPARD, PRICKLE3 and WNT2B. 9 up-regulated DElncRNAs and 8 down-regulated DElncRNAs were involved in regulation. Among them, WNT5A, WNT2B, WNT10B, WNT16 and FZD3 were expressed at higher levels in IMF. PPARD was expressed at higher levels in SCF, and FOSL1. Although differentially expressed in both adipose tissues, the expression levels were lower than other genes. Wnt signaling pathway was divided into classical Wnt signaling pathway and atypical Wnt signaling pathway (38). The typical Wnt signaling pathway enhances preadipocyte proliferation (39) and inhibits preadipocyte to adipocyte differentiation (40). The atypical Wnt signaling pathway inhibits PPAR transcription in bone marrow mesenchymal stem cells, thereby suppressing the differentiation of mesenchymal stem cells into adipocytes (41). WNT family proteins are involved in a variety of biological regulatory processes, including cell differentiation, proliferation and migration, embryogenesis, and cardiovascular development. Most WNT family proteins (WNT10B) activate the typical Wnt signaling pathway, and a few proteins (WNT5A) activate the atypical Wnt signaling pathway. FDZ3 is a transmembrane receptor that preferentially transduces Wnt signaling based on ligand dependence. WNT5A inhibits PPARγ expression and suppresses adipogenesis by activating the atypical Wnt signaling pathway (42). In addition, increased WNT5A expression leads to inflammation and glucose tolerance (43). WNT5A can also involve in the MAPK signaling pathway to inhibit mid- to late-stage differentiation during adipose differentiation (44). WNT10B increases insulin resistance in mature adipocytes, resulting in impaired insulin transmission and reduced glucose uptake (45). In addition to involve in the Wnt signaling pathway, FOSL1 can inhibit adipocyte differentiation by inhibiting C/EBPα (46). FOSL1 is a potential therapeutic target to prevent diabetes-induced atherosclerosis (47). The existing literatures showed that lncRNAs, such as TCONS_00006525, TCONS_00046551, and TCONS_00000528 in the regulatory network are involved in the WNT signaling pathway. They can to inhibit adipogenesis and lipid metabolism in IMF tissues by regulating genes such as WNT5A, WNT10B, and FDZ3. The prediction of lncRNA binding to protein showed the potential of TCONS_00006525 to participate in the protein level regulation.

In the lncRNA-miRNA-mRNA regulatory network, GO annotation analysis showed that the target genes were significantly enriched in biological functions such as signaling pathway regulation, cell biofilm composition and transcription factor activity regulation; KEGG through-link analysis showed that the target genes were mainly involved in PPAR, TNF, Wnt and other signaling pathways. In this study, 3 differential genes were significantly enriched in the PPAR signaling pathway, including RXRG, SCD, and PPARD. The osmoproliferator-activated receptor family(PPARs) are lipid activators that play multiple roles in adipogenesis and metabolism. The main function of PPARD is to regulate adipogenesis and lipid metabolism directly or by activating related genes. In mice, PPARD agonists alleviate obesity induced by a high-fat diet. The high expression of activated PPARD in adipocytes promotes fatty acid oxidation and utilization in *in vitro* experiments (48). PPARD can form a complex protein with the retinoid X receptor (RXR) then bind to the peroxisome proliferator response element (PPRE), thereby activating the expression of a variety of genes involved in lipid metabolism (49). In addition, PPARD interacts with β-catenin and assists β-catenin in the induction of gene expression (49). Stearoyl coenzyme A desaturase (SCD) is a key enzyme in fatty acid metabolism, catalyzing the production of unsaturated fatty acids and participating in leptin metabolism (50). In the subcutaneous fat of Laiwu and Daibai pigs (typical lean pigs), here is a significant difference in the expression level of SCD. This is consistent with the results of this study (51). In this study, TCONS_00026517 and other lncRNAs were involved in the PPARA and Wnt signaling pathways to regulate lipid metabolism by regulating the expression levels of PPARD, RXRG, and SCD through the ceRNA network.

## 5 Conclusions

In this study, a total of 1258 differentially expressed lncRNAs (DElncRNAs) were identified in IMF and SCF, including 592 up-regulated and 666 down-regulated lncRNAs. The analysis of the DElncRNA co-expression network and ceRNA network showed that TCONS_00006525, TCONS_ 00026517 and other lncRNAs regulated adipogenesis and lipid metabolism in IMF and SCF regulate biological processes such as signaling pathways, biofilm composition, transcription factor activity and Wnt, PPAR and TNF signaling pathways.

## Data availability statement

The original contributions presented in the study are publicly available. RNA-Seq data have been deposition in the NCBI Sequence Read Archive (SRA) database with accession number PRJNA870052 and PRJNA870173.

## Ethics statement

The animal study was reviewed and approved by Animal Care Commission of the Institute of Animal Sciences, Chinese Academy of Agricultural Sciences.

## Author contributions

XM conceived, designed and performed the experiments and wrote the paper; HF performed the experiment and data analysis and wrote the paper; TL, SY, XZ, WH, AL, and LX performed the experiments. All of the authors read and approved the final manuscript.
